# What can we learn about perpetrators of domestic and familial homicide and their involvement with mental health services from domestic homicide reviews?

**DOI:** 10.1192/j.eurpsy.2021.1014

**Published:** 2021-08-13

**Authors:** P. Macinnes

**Affiliations:** Ioppn, King’s College London, London, United Kingdom

## Abstract

**Introduction:**

The evidence that supports an association between domestic violence and abuse (DVA) perpetration and mental disorders is increasing. Since 2011, authorities in England and Wales have been required to conduct Domestic Homicide Reviews (DHRs) into deaths caused by violence, abuse or neglect of individuals aged 16 or over, by a family member or a current or ex-partner.

**Objectives:**

The aim of the study is to describe the characteristics of perpetrators of domestic homicide in a sample of DHR reports in which the perpetrator was known to mental health services in the 12 months before the offence. This sample will undergo qualitative framework analysis as part of another study conducted by the authors.

**Methods:**

The researchers compiled a list of DHRs available online and randomly sampled 168 reports; in 20 of those reports, the perpetrators were under the care of mental health services in the 12 months prior to the offence. We have applied descriptive statistics to report on the sample characteristics.

**Results:**

The common mental illnesses diagnosed amongst perpetrators were depression (20%), anxiety (15%) and schizophrenia (15%). 25% of the perpetrators didn’t have a psychiatric diagnosis and 70% had a history of self-harm and suicidal ideation. Substance use was prevalent (60%). Half of the homicides involved children.

**Conclusions:**

This report describes the demographic and mental health characteristics of a sample of perpetrators of domestic homicides. Further research is needed into the patterns of mental health service use by DVA perpetrators in order to improve identification and risk management.

**Conflict of interest:**

No significant relationships.

